# Parenting as a Moderator of the Relation Between Child Inhibited Temperament and Anxiety in Western Contexts: A Systematic Review

**DOI:** 10.1007/s10567-024-00492-5

**Published:** 2024-06-16

**Authors:** Elizabeth M. Aaron, Emma Caley, Elizabeth J. Kiel

**Affiliations:** Psychology Department, Miami University, 90 N Patterson Ave, Oxford, OH 45056 USA

**Keywords:** Inhibited temperament, Behavioral inhibition, Parenting, Anxiety

## Abstract

The literature on the role of parenting in the relation between child inhibited temperament and child anxiety is inconsistent, with some literature supporting a moderating role and some literature supporting alternative (e.g., mediating) roles. A systematic review of the evidence that parenting moderates the longitudinal relation between child inhibited temperament and child anxiety is needed. A systematic review of the literature was conducted in February and March of 2022 and repeated in January of 2024. Ten articles met criteria for inclusion, with 39 moderation analyses of interest among them. All included studies were conducted in Western contexts with predominately White, middle-class families. Thus, the current review can only be generalized to this population. Despite inconsistent findings, some evidence indicated that avoidance-promoting parenting behaviors such as overprotection and overinvolvement moderate the relation between child inhibited temperament and social anxiety symptoms, in particular. There was a lack of evidence that parenting behaviors moderate the relation between child inhibited temperament and anxiety disorders, and that affect-related parenting behaviors (e.g., negativity) moderate the relation between child inhibited temperament and non-social anxiety symptoms. There was mixed evidence regarding the moderating role of control-related parenting behaviors in the relation between child inhibited temperament and non-social anxiety symptoms, with some evidence that encouraging behaviors moderate this relation. Future research is needed to clarify these inconsistent and nuanced findings and investigate this moderation in non-Western, non-White, and low-income populations.

## Introduction

Inhibited temperament, or behavioral inhibition, is a temperamental dimension defined by withdrawal and hesitancy in novel environments (Kagan et al., 1984). Children with more inhibited temperaments have a higher likelihood of developing anxiety (Dyson et al., 2011; Sandstrom et al., 2020). Further, child inhibited temperament is most strongly linked to the development of social anxiety, specifically (Biederman et al., 2001; Chronis-Tuscano et al., 2009; Clauss & Blackford, 2012). Although many studies have found a relation between inhibited temperament and child anxiety, some studies have found that this relation only occurs in certain contexts (Crockenberg & Leerkes, 2006; White et al., 2017). One context that has been widely studied is that of the parenting environment. Certain parenting behaviors have been linked to child anxiety, including control-related parenting behaviors (e.g., overcontrol, overprotection, encouragement, intrusion; Edwards et al., 2010a) and negative affect-related parenting behaviors (e.g., expressed anxiety, negativity, criticism; McLeod et al., 2007). There is research to suggest that control- and affect-related parenting behaviors moderate the relation between inhibited temperament and child anxiety, such that children who have more inhibited temperaments are more likely to develop anxiety only in the context of receiving high levels of these parenting behaviors (Lewis-Morrarty et al., 2012; Lorenzo et al., 2022). In other words, children who exhibit more fearfulness and withdrawal in novel situations in the context of receiving parenting that criticizes or reinforces these behaviors are more likely to develop child anxiety.

The moderating role of parenting in the relation between inhibited temperament and anxiety has been a major focus within the field, and yet it has not been reviewed systematically. Without a systematic review of the literature, the strength of research evidence for this moderating effect is unknown. Indeed, some research has not found evidence of parenting moderating the relation between inhibited temperament and anxiety (Hudson et al., 2011; Muris et al., 2011). Further, research suggests that parenting may have a different role than as a moderator in this relation. A recent literature review provided support for a relation between inhibited temperament and child anxiety, and then proposed a model of the developmental pathways from inhibited temperament to anxiety that included parenting as both a moderator and a mediator (Liu & Pérez-Edgar, 2019). Given the emerging research on parenting serving alternative roles, it is important to determine if there is sufficient evidence to continue modeling parenting as a moderator. The current review builds on the foundation of Liu and Pérez-Edgar (2019) by systematically investigating the previous research on parenting serving as a moderator.

### Inhibited Temperament and Child Anxiety

Temperament is defined as a relatively stable biologically based dimension reflecting individuals’ typical emotional, physiological, attentional, and regulatory responses to their environment (Goldsmith et al., 1987; Shiner et al., 2012). Inhibited temperament (also referred to as behavioral inhibition or fearful temperament) is characterized by fearful, shy, and withdrawn responses to new stimuli and environments (Kagan et al., 1984). Behavioral inhibition is assessed both dichotomously (including children with extreme inhibited vs. uninhibited behavior only) and dimensionally (assessing all children’s varying levels of inhibition). Children with high inhibited temperament are more physiologically reactive to their environments, have more attentional bias toward novelty, have more difficulty shifting their attention away from threat, and are more likely to interpret ambiguous environmental stimuli as threatening (Pérez-Edgar & Guyer, 2014). An infant with a high inhibited temperament may move away from a stranger or an unknown toy or cry in response to these stimuli. These inhibited behaviors mirror behaviors seen in later child anxiety, when children exhibit fear and withdrawal in the presence of anxiety-provoking stimuli. Indeed, previous research has demonstrated that inhibited temperament is the strongest predictor of later child anxiety (Biederman et al., 2001; Buss et al., 2021; Clauss & Blackford, 2012; Liu & Pérez-Edgar, 2019). Inhibited temperament is most strongly linked to child social anxiety (Biederman et al., 2001; Buss et al., 2021; Clauss & Blackford, 2012). However, it is also related to other anxiety disorders such as generalized anxiety disorder and specific phobia (Sandstrom et al., 2020). These differing relations may be explained by the context in which inhibited temperament is measured, with measurement in social environments mapping more onto social anxiety symptoms and measurement in non-social environments relating more to other anxiety symptoms (Dyson et al., 2011; Tan et al., 2024).

The relation between inhibited temperament and anxiety is consistent with theories of anxiety development which posit that biological vulnerabilities, such as inhibited temperament, serve as predisposing risk factors for the development of anxiety (Barlow, 2000; Muris, 2006). These theories also acknowledge the important role of a child’s environment in the development of later psychopathology, considering environmental factors such as parenting as predisposing factors that interact with child temperament (Muris, 2006). Therefore, it is important to consider the role of the environment in the development of child anxiety.

### Parenting and Child Anxiety

Parents and caregivers are integral to children’s development, and often regulate the manner in which children engage with their environments. Indeed, family systems theory indicates that the way in which family members interact with one another influences the development of individual family members and the family system itself. Within the realm of parenting, family systems theory suggests an anxious-coercive cycle in which children with high inhibited temperaments elicit overprotective and overcontrolling behaviors from their caregivers, which then serve to reinforce the child’s inhibition (Dadds & Roth, 2001). When a caregiver provides a child with high amounts of comfort and reassurance or seeks to control children’s behavior when faced with low-threat environmental stimuli, such as a friendly stranger or safe toy, this behavior can communicate to the child that they need their caregiver to help them navigate these stimuli.

Overprotective behaviors such as comfort and reassurance hinder child engagement with novel stimuli, and thus can be categorized as control-related behaviors that promote child avoidance. In other words, when parents comfort their children and attempt to protect them from unfamiliar environments, it reinforces children’s withdrawal and prevents them from engaging with the environment. It is well documented that anxiety is reinforced by avoidance of feared stimuli, and treated through exposure to feared stimuli (Peris et al., 2017; Whiteside et al., 2013, 2020). Thus, when parents promote avoidance and limit exposure to these stimuli, they feed an anxious cycle for their inhibited children.

Alternatively, overcontrolling behaviors such as intrusive directives seek to control children’s engagement with unfamiliar stimuli, and thus can be categorized as control-related behaviors that excessively promote approach. When parents dictate how children should engage with their environments, children’s autonomy is reduced. Behaviors within both of these control-related categories contribute to children feeling less in control of their environments. When children have low self-efficacy regarding their ability to independently manage their environments, they are more likely to develop anxiety (Chorpita & Barlow, 1998).

Other parenting behaviors have been linked to child anxiety as well, including affect-related parenting behaviors such as negativity, dismissiveness, and criticism, which are characterized by negative affect and rejection (Gouze et al., 2017; McLeod et al., 2007). When caregivers respond to their children with high amounts of negative affect, these responses may limit children’s beliefs in their own capabilities, engendering the same feeling of a lack of control that is theorized to relate to child anxiety (Chorpita & Barlow, 1998; Gouze et al., 2017). Additionally, when caregivers respond to their children with criticism, it may cause their children to perceive social interactions as more threatening due to an expectation of further criticism, which then perpetuates anxiety (Garcia et al., 2021). Although the anxious-coercive cycle focuses on avoidance-promoting and rejecting parenting behaviors, the potential impact of negativity and criticism on children’s self-efficacy beliefs may create a similar cycle that maintains both the child temperamental risk for anxiety and the anxiogenic parenting risk for anxiety (Dadds & Roth, 2001). The anxious-coercive cycle highlights the joint role of parent and child factors in child anxiety development, and the way in which overcontrolling parenting behaviors engender child anxiety, both of which will be investigated in the current review. Previous research also suggests that parenting may serve as a contextual factor in the development of child social anxiety and other types of child anxiety.

### Parenting as a Moderator

The trajectory of child anxiety development is influenced by child inhibited temperament and parenting behavior (Biederman et al., 2001; Edwards et al., 2010a). Previous research has investigated the interaction between these variables in the development of child anxiety. It has been posited that children with high levels of inhibited temperament only go on to develop childhood social withdrawal and anxiety when their parents engage in high levels of anxiogenic parenting behaviors, such as overcontrolling and critical parenting (Liu & Pérez-Edgar, 2019; Rubin et al., 2009). This theory has been supported by previous research that has investigated numerous parenting behaviors as moderators and both social and non-social anxiety as outcomes (Kiel et al., 2016; Lewis-Morrarty et al., 2012; Lorenzo et al., 2022). For example, Lewis-Morrarty et al. (2012) found that stable childhood behavioral inhibition only predicted adolescent social anxiety within the context of mothers’ high levels of overcontrol. However, there are some studies that have failed to find a significant moderating effect of parenting behavior in this relation (Hudson et al., 2011; Muris et al., 2011). Hudson et al. (2011) found that children with behavioral inhibition (dichotomously assigned) were more likely to have a diagnosis of social phobia and generalized anxiety disorder, and that parenting did not moderate this relation.

Only some of these previous moderation studies used rigorous prospective designs to assess parenting as a moderator within the directional relation from child inhibited temperament to child anxiety. Some of the previous research was conducted concurrently, which limits the interpretability of these moderation results. Given the mixed findings and the inconsistent methodological rigor in the previous literature, it is important to conduct a systematic review to elucidate whether there is substantial evidence of parenting serving as a moderator of the relation between child inhibited temperament and child anxiety. Notably, the presence of moderation can be evaluated by assessing either parenting or inhibited temperament as the moderator (and the other variable as the predictor). The current review will include articles that assess this interaction, no matter the assignment of roles.

It is also important to note the various theoretical frameworks in which this moderation can be assessed. The interaction between child inhibited temperament and parenting can be conceptualized within a diathesis-stress model, a vantage-sensitivity model, or a differential susceptibility model. Within a diathesis-stress framework, child inhibited temperament would be considered a risk factor, and anxiogenic parenting behavior would be considered a stressor that, in interaction with inhibited temperament, predicts anxiety outcomes (Zuckerman, 1999). Within a differential susceptibility framework, children with high inhibited temperaments would be expected to be more negatively impacted by anxiogenic parenting and more positively impacted by adaptive parenting in comparison to children with moderate or low levels of inhibited temperament (Belsky & Pluess, 2009). Lastly, within a vantage-sensitivity framework, children with higher inhibited temperaments would have less anxiety within the context of adaptive parenting behaviors as compared to children with moderate or low levels of inhibited temperament (Pluess & Belsky, 2013). The current review will assess the foundational models used in the included articles. The paper will not weigh the evidence for each of these models, but instead will discuss how these theoretical frameworks may impact the moderation findings in the literature.

### Objectives of the Review

The current state of the research on inhibited temperament and child anxiety indicates that it is time to examine whether there is sufficient research evidence to assert that parenting serves a moderating role in the relation between inhibited temperament and child anxiety. The current paper will systematically review the previous literature, assess the methodological rigor and quality of the included studies, and lastly, evaluate and integrate the included studies’ findings to elucidate the strength of the evidence for parenting moderating the relation between child inhibited temperament and child anxiety, considering both type of anxiety (social or other type) and the type of parenting assessed.

## Method

### Literature Review

A systematic review of the literature was conducted in February and March of 2022, and then repeated in January of 2024. PsycINFO, MedLine, and PubMed were searched in February 2022 and January 2024 to find relevant articles. The following search terms were used: (moderat* or interact*) AND (parenting or mother–child relations or parent–child relations or child rearing or family) AND (inhibited temperament or inhibit* or fearful temperament or behavioral inhibit* or dysregulated fear) AND (anxi* or wariness or withdrawal or reticence or internaliz*) NOT (mice or rat or rodent* or mouse or autis* or asd or pharmac*). Only academic journal articles written in English were included in the search results. For MedLine and PsycINFO, the search was expanded to the full-text for the terms (moderat* or interact*). For all of the PubMed search terms and the rest of the MedLine and PsycINFO search terms, the detailed record was searched (i.e., title, abstract, keywords, subject headings). Once these searches were completed, the articles were exported to Zotero and duplicates were merged. Next, the first author screened the titles of all articles and removed articles with titles that did not refer to at least one of the pertinent review topics (i.e., temperament, parenting, and anxiety). Next, the first author screened the remaining abstracts and removed articles that did not mention all of the following topics: temperament, environment/parenting, and anxiety/anxiety-relevant domain. Lastly, the first and second authors screened the remaining full-text articles to determine which articles met the review inclusion criteria. The inter-rater reliability of the eligibility decisions made by the authors was κ = 0.64, which is considered moderate reliability (McHugh, 2012). Discrepancies were discussed and consensus was reached to finalize the list of included articles. Lastly, the first author screened the reference sections of included articles. Full-text articles identified in the reference sections were screened for eligibility and added to the list of included articles if they met all criteria.

### Inclusion and Exclusion Criteria

Articles were included in the review if they met the following inclusion criteria: based in a laboratory context; assessed child inhibited temperament or a related temperamental dimension such as fearful temperament or behavioral inhibition when children were 6 years of age or younger; assessed child (children ages 17 years and younger) anxiety at least 6 months after temperament was assessed; assessed parenting behavior; examined the moderating role of parenting in the prospective relation between inhibited temperament and child anxiety or the moderating role of inhibited temperament in the prospective relation between parenting and child anxiety. Articles were excluded from the review if they were not written in English, were dissertations, or focused on children with autism spectrum disorder, other developmental disabilities, or chronic health conditions.

### Study Selection

**Fig. 1:**
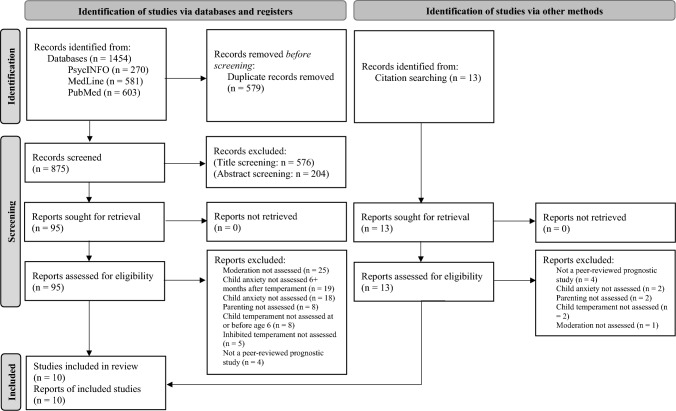
Search strategy and results

Ten articles including eight samples were identified for inclusion in the current review. The articles were published between 2011 and 2021. Details of the articles reviewed at each screening stage are located in Fig. 1. One hundred and eight full-text articles were assessed for eligibility, 95 from databases and 13 from citation searches. The primary reasons for exclusion during the full-text review were that the moderation of interest was not assessed, child anxiety was not assessed at least 6 months following the assessment of child inhibited temperament, and that child anxiety was not assessed. Other reasons for exclusion are found in Fig. 1. Three of the articles included in the review (Hudson & Dodd, 2012; Hudson et al., 2011, 2019) used the same sample of participants with the same baseline data. However, each article used a different timepoint for measuring child anxiety at time 2, thus providing unique longitudinal results regarding the study question. Thus, all three articles were included in the present review. See Table 1 for a description of the included studies.

**Table 1:** Study descriptions

Study	*N*, race/ethnicity, socioeconomic status, sample	Child sex or gender	Child inhibited temperament	Parenting	Child anxiety	Results of moderation analyses	Risk of bias score
Hudson & Dodd, 2012	*N* = 202 *Race/ethnicity:* 64% Oceanic, 20% European, 10% Asian, 6% American, African or Middle Eastern *SES:* 56% middle to high income; 42% of mothers worked part-time; 92% of mothers completed high school; 85% of mothers had a post-school qualification *Sample:* Australia, infants from community identified as behaviorally inhibited or behaviorally uninhibited	*Gender*: 50% boys at baseline	*Measure:* Short Temperament Scale for Children, approach scale; Observed BI during novel tasks similar to Garcia Coll et al., 1984 *Child Age:* 4 years	*Measure:* Observed overinvolvement and negativity during speech preparation task and the Five-Minute Speech Sample; Parent Protection Scale, Control scale *Child Age:* 4 years	*Measure:* Presence of anxiety disorder diagnosis and number of anxiety diagnoses from the ADIS-IV *Child Age:* 4 and 9 years	4 non-significant moderation analyses	5
Hudson et al., 2011	*N* = 202 *Race/ethnicity:* Behaviorally inhibited children: 61% Australian, 17% Asian, 17% European, 7% other Behaviorally uninhibited children: 69% Australian, 3% Asian, 21% European, 7% other *SES:* Behaviorally inhibited children: 58% middle to high income; Maternal highest education: 14% school, 29% post-school, 57% degree Behaviorally uninhibited children: 59% middle to high income; Maternal highest education: 17% school, 40% post-school, 43% degree *Sample:* Australia, infants from community identified as behaviorally inhibited or behaviorally uninhibited	*Sex*: 50% male	*Measure:* Short Temperament Scale for Children, approach scale; Observed BI during novel tasks similar to Garcia Coll et al., 1984 *Child Age:* 4 years	*Measure:* Observed overinvolvement and negativity during speech preparation task and the Five-Minute Speech Sample; Parent Protection Scale, Control scale *Child Age:* 4 years	*Measures:* Presence of anxiety disorder diagnosis and number of anxiety diagnoses from the ADIS-IV; PAS *Child Age:* 4 and 6 years	4 non-significant moderation analyses	3
Hudson et al., 2019	*N* = 202 *Race/ethnicity:* Behaviorally inhibited (BI) children: 57% Australian/Caucasian, 21% Asian, 12% European, 3% American, 3% African, 3% Middle Eastern Behaviorally uninhibited (BUI) children: 79% Australian/Caucasian, 5% Asian, 12% European, 1% American, 1% African, 2% Middle Eastern *SES:* Behaviorally inhibited family income: 76% > $80 k, 19% $40 k-80 k, 6% < 40 k Behaviorally uninhibited family income: BUI: 71% > $80 k, 17% $40-80 k, 11% < 40 k *Sample:* Australia, infants from community identified as behaviorally inhibited or behaviorally uninhibited	*Gender*: BI children: 51% girls; BUI children: 49% girls	*Measure:* Short Temperament Scale for Children, approach scale; Observed behavioral inhibition during novel tasks similar to Garcia Coll et al., 1984 *Child Age:* 4 years	*Measure:* Observed overinvolvement and negativity during speech preparation task and the Five-Minute Speech Sample; Parent Protection Scale, Control scale *Child Age:* 4 years	*Measures and Child Age:* Presence of anxiety disorder diagnosis from the ADIS-IV at child ages 4, 6, 9, and 12 years; PAS at child ages 4 and 6 years; Spence Children’s Anxiety Scale at child ages 9 and 12 years	1 significant and 5 non-significant moderation analyses	4
Kiel et al., 2016	*N* = 117 *Race/ethnicity:* Mothers and toddlers, respectively: 97 (88.2%) and 92 (83.6%) European American, 2 (1.8%) and 6 (5.5%) African American, 7 (6.4%) and 9 (8.2%) Asian American, 1 (0.9%) and 1 (0.9%) American Indian, and 3 (2.8%) and 2 (1.8%) “other ethnic/racial identity.” In addition, one (0.9%) mother and six (5.5%) toddlers were identified as Hispanic/Latino *SES:* According to the Hollingshead four-factor index, participants were on average middle-class *Sample:* United States, community	*Gender*: 44% girls	*Measure:* observed IT during novelty tasks *Child Age:* 2 years	*Measure:* Maternal encouragement to approach novelty during novelty tasks *Child Age:* 2 years	*Measure:* Infant Toddler Social-Emotional Assessment-Revised separation distress subscale *Child Age:* 2 and 3 years	1 significant moderation analysis	3
Lawrence et al., 2020	*N* = 217 *Race/ethnicity:* Mostly Caucasian *SES:* Control group: 82.5% middle/upper class Social anxiety disorder (SAD) group: 73.3% middle/upper class Generalized anxiety disorder (GAD) group: 81.6% middle/upper class *Sample:* United Kingdom, mothers from community pre-screened for inclusion based on if they met criteria for social anxiety disorder, generalized anxiety disorder, or neither disorder	*Sex:* Control group: 51% female; SAD group: 64% female; GAD group: 57% female	*Measures and Child Age:* Observation of negative reactivity during Kagan et al., 1987 novelty tasks at child age 4 months; observation of BI during novelty tasks at child age 14 months	*Measure:* Observation of maternal encouragement, expressed anxiety, and intrusiveness during novelty tasks *Child Age:* 10 and 58 months	*Measures:* Presence of social anxiety disorder diagnosis from the ADIS-IV; CBCL anxiety problems scale *Child Age:* 58 months	1 significant and 5 non-significant moderation analyses	6
Lewis-Morrarty et al., 2012	*N* = 176 *Race/Ethnicity:* 100% European American *SES:* Middle-to-upper class; 28% of mothers graduated high school, 49% graduated college, 11% completed graduate school *Sample:* United States, community	*Gender:* 51% girls	*Child Ages 1 and 2 years*: Observed BI during Calkins et al., 1996, Fox et al., 2001, and Kagan et al., 1987 tasks; Toddler Behavior Assessment Questionnaire, Social Fearfulness scale *Child Ages 4 and 7 Years*: Play Observation Schedule while playing with peers and Colorado Children's Temperament Inventory, Shyness/Sociability scale	*Measure:* Observation of maternal overcontrol during Hane et al., 2008 tasks *Child Age:* 7 years	*Measure:* Presence of lifetime diagnosis of social anxiety disorder from K-SADS and additional probe questions from the ADIS; SCARED social anxiety subscale *Child Age:* 14–17 years	1 significant and 1 marginally significant moderation analysis	3
Lorenzo et al., 2022	*N* = 291 *Race/ethnicity:* 69.1% White, 16.5% Black, 7.2% Hispanic, 3.1% Asian, 3.4% other, and < 1% missing *SES:* 77.6% of parents had 2- or 4-year college degree *Sample:* United States, infants from community screened for higher levels of negative and positive reactivity to novelty at 4 months	*Sex*: 55% female	*Measure:* Observed temperament during Fox et al., 2001 novelty tasks *Child Age:* 36 months	*Measure:* Observation of dismissive, task- directive, and supportive parenting during LabTAB episodes *Child Age:* 36 months	*Measures and Child Age:* SCARED social anxiety subscale at child ages 9, 12, and 15 years controlling for CBCL anxiety problems domain at child age 4 years	3 significant and 3 non-significant moderation analyses	7
Majdandžić et al., 2018	*N* = 132 *Race/ethnicity:* 89% of mothers and 95% of fathers had Dutch origin *Education:* Scale from 1 = primary education to 8 = university education Maternal Education: *M* = 7.05, range = 1–8 Paternal Education: *M* = 6.57, range = 2–8 *Professional Level:* Scale from 1 = manual labor to 11 = labor for which a university degree is required Maternal Professional Level: *M* = 8.70, range = 2–11 Paternal Professional Level: *M* = 8.22, range = 3–11 *Sample:* Netherlands, first-time community parents	55% girls^a^	*Measures and Child Age:* Observed negative reactivity during Kagan & Snidman, 1991 novelty tasks at child age 4 months; observed fearful temperament during Aktar et al., 2013 novelty tasks at child age 1 year	*Measure:* Observed challenging and overprotective parenting behavior during structured tasks and unstructured free play *Child Age:* 1 and 2.5 years	*Measure:* PAS-R *Child Age:* 2.5 and 4.5 years	1 marginally significant and 3 non-significant moderation analyses	6
Murray et al., 2014	*N* = 136 *Race/ethnicity:* Control group: 98% White; Social anxiety group: 100% White *SES:* Control group: 73% upper class; Social anxiety group: 68.5% upper class *Sample:* United Kingdom, mothers from community pre-screened for inclusion based on if they met criteria for social anxiety disorder or neither social nor generalized anxiety disorder (control)	*Sex:* Control group: 47.6% boys; Social anxiety group: 39.7% boys	*Measure:* Observed BI during Kagan et al., 1987 novelty tasks *Child Age:* 14 months	*Measure:* Maternal encouragement and attribution of threat to the environment while reading a book about going to school to their child *Child Age:* 4–5 years	*Measure:* Presence of social anxiety disorder diagnosis from the ADIS-IV *Child Age:* 4–5 years	2 non-significant moderation analyses	8
Vreeke et al., 2013	*N* = 168 *Race/ethnicity:* 53% Native Dutch children who were all White/Caucasian and had parents and grandparents who were born in the Netherlands. 47% Non-Native Dutch children who were offspring of immigrant families originating from a variety of countries (11.9% from Surinam and the Netherlands Antilles, 2.4% from Morocco, 4.2% from Turkey, and 27% from other countries) *SES:* Not reported *Sample:* Netherlands, community	*Sex:* 54% boys	*Measure:* Short version of the Behavioral Inhibition Questionnaire *Child Age:* 3–6 years	*Measure:* The Parental Overprotection Measure *Child Age:* 3–6 and 4–7 years	*Measure:* PAS-R social anxiety subscale and composite of other anxiety subscales *Child Age:* 3–6 and 4–7 years	1 significant and 3 non-significant moderation analysis	11

*ADIS-IV* Anxiety Disorder Interview Schedule for DSM-IV, *BI* behavioral inhibition; *CBCL* Child Behavior Checklist; *IT* inhibited temperament; *K-SADS* Kiddie Schedule for Affective Disorders and Schizophrenia for School-Age Children-Present and Lifetime Version; *PAS* Preschool Anxiety Scale; *PAS-R* Preschool Anxiety Scale-Revised; *SCARED* Screen for Child Anxiety Related Emotional Disorders

^a^The terms sex and gender were not used to describe the labels used in this sample

### Sample Characteristics

The eight samples included in the current review were relatively racially and ethnically homogenous, with all samples comprised of a majority of White, non-Hispanic participants. There was minimal diversity in the geographic location of the eight samples, as well, including the United States (*n* = 3), the Netherlands (*n* = 2), the United Kingdom (*n* = 2), and Australia (*n* = 1). Given that all of these studies had Western samples with primarily White, non-Hispanic participants, this review will only reflect the evidence for moderation in Western contexts. Most samples were majority middle-to-upper class. All study samples were recruited from the community. One sample pre-screened community children for high and low levels of inhibited temperament for inclusion (Hudson & Dodd, 2012; Hudson et al., 2011, 2019). One sample pre-screened community children for high positive and high negative reactivity to novelty as infants, and oversampled these infants for inclusion (Lorenzo et al., 2022). One sample pre-screened community mothers, and included mothers who met criteria for generalized anxiety disorder or social anxiety disorder and mothers who did not meet criteria for either disorder (Lawrence et al., 2020). One sample pre-screened pregnant mothers, and included mothers who met criteria for social anxiety disorder and mothers who did not meet criteria for social anxiety disorder or generalized anxiety disorder (Murray et al., 2014). One sample recruited first-time parents (Majdandžić et al., 2018). All other samples recruited community families with no pre-screening conducted for inclusion. All study samples had an approximately equal breakdown in binary designations of child sex or gender (no studies reported separate demographics for sex and gender, and no studies included children with non-binary identities). Samples ranged in size from 117 to 291 families.

Across the samples, child inhibited temperament was first assessed when children were between the ages of 4 months and 6 years. Three samples (Lawrence et al., 2020; Majdandžić et al., 2018; Murray et al., 2014) assessed inhibited temperament when children were infants (0–15 months), one sample (Kiel et al., 2016) assessed inhibited temperament when children were toddlers (16–35 months), three samples (Hudson & Dodd, 2012; Hudson et al., 2011, 2019; Lorenzo et al., 2022; Vreeke et al., 2013) assessed inhibited temperament when children were pre-school or school-aged (3–6 years), and one sample created a longitudinal inhibited temperament profile using assessments from when children were 14 months, 24 months, 4 years, and 7 years (Lewis-Morrarty et al., 2012). Parenting behaviors were first assessed when children were between the ages of 10 months and 6 years. One sample (Lawrence et al., 2020) assessed parenting when children were infants (0–15 months), one sample (Kiel et al., 2016) assessed parenting when children were toddlers (16–35 months), one sample assessed parenting when children were infants and toddlers (at 1 and 2.5 years old; Majdandžić et al., 2018), and five samples (Hudson & Dodd, 2012; Hudson et al., 2011, 2019; Lewis-Morrarty et al., 2012; Lorenzo et al., 2022; Murray et al., 2014; Vreeke et al., 2013) assessed parenting when children were pre-school or school-aged (3–7 years). Child anxiety was assessed as the outcome when children were between the ages of 3 and 17 years. Because each article by Hudson and colleagues assessed child anxiety at a different age, this sample will be broken down by article. Most articles (*n* = 7; Hudson & Dodd, 2012; Hudson et al., 2011; Kiel et al., 2016; Lawrence et al., 2020; Majdandžić et al., 2018; Murray et al., 2014; Vreeke et al., 2013) assessed anxiety when children were in early to middle childhood (2–9 years). Two articles (Hudson et al., 2019; Lorenzo et al., 2022) assessed anxiety over time from early to middle childhood (3–9 years) through adolescence (12–15 years) and one article (Lewis-Morrarty et al., 2012) assessed anxiety in adolescence (14–17 years). The time between the measure of inhibited temperament and the measure of anxiety ranged from 1 to 16 years, with most studies assessing them 1–5 years apart (*n* = 7; Hudson & Dodd, 2012; Hudson et al., 2011; Kiel et al., 2016; Lawrence et al., 2020; Majdandžić et al., 2018; Murray et al., 2014; Vreeke et al., 2013).

### Method Characteristics

#### Measures of Inhibited Temperament

Inhibited temperament was measured in a variety of ways across the study samples. Inhibited temperament was measured dimensionally in five samples (Kiel et al., 2016; Lewis-Morrarty et al., 2012; Lorenzo et al., 2022; Majdandžić et al., 2018; Vreeke et al., 2013) and dichotomously in three samples (Hudson & Dodd, 2012; Hudson et al., 2011, 2019; Lawrence et al., 2020; Murray et al., 2014). Most samples used an observational measure (*n* = 7; Kiel et al., 2016; Hudson & Dodd, 2012; Hudson et al., 2011, 2019; Lawrence et al., 2020; Lewis-Morrarty et al., 2012; Lorenzo et al., 2022; Majdandžić et al., 2018; Murray et al., 2014), with two of these samples also using a parent-report measure. One sample used only a parent-report measure (Vreeke et al., 2013). All seven samples with observational measures used standardized procedures (from Aktar et al., 2013; Calkins et al., 1996; Fox et al., 2001; Kagan et al., 1987; Kagan & Snidman, 1991; Rubin, 2001) in which infants and children interacted with novel stimuli, such as unknown toys and people, and were coded for inhibited and fearful behaviors, such as distress vocalizations and proximity to caregiver.

The three samples that utilized parent-report measures of temperament all used different scales. The Hudson and colleagues sample (Hudson & Dodd, 2012; Hudson et al., 2011, 2019) used the approach scale of the Short Temperament Scale for Children, using one standard deviation or more below the mean to represent uninhibited temperament and one standard deviation or more above the mean to represent inhibited temperament (Sanson et al., 1994). Lewis-Morrarty et al. (2012) used the Social Fearfulness scale of the Toddler Behavior Assessment Questionnaire (Goldsmith, 1996) in addition to the Shyness/Sociability subscale of the Colorado Children's Temperament Inventory (Buss & Plomin, 1984; Rowe & Plomin, 1977). Vreeke et al. (2013) used the short version of the Behavioral Inhibition Questionnaire (Bishop et al., 2003; Edwards, 2007).

Four of the eight study samples used more than one measure of inhibited temperament. The Hudson and colleagues sample (Hudson & Dodd, 2012; Hudson et al., 2011, 2019) used the Short Temperament Scale for Children (Sanson et al., 1994) for their main analyses. Then, they re-ran their analyses with the behaviorally inhibited group only including children who met the cutoff for inhibition on both the observational and parent-report measures. Lewis-Morrarty et al. (2012) conducted a latent profile analysis using data from all of their parent-report and observational measures in order to create a continuous variable of the probability that each child would belong to the high behavioral inhibition profile. Lawrence et al. (2020) used a dichotomous measure of stability in inhibited temperament through identifying children who were categorized as negatively reactive at time 1 and behaviorally inhibited at time 2. Majdandžić et al. (2018) used both time 1 and time 2 assessments of fearful temperament in their analyses.

#### Measures of Parenting

Various parenting dimensions related to control and negative affect were examined in the included articles. Most of the studies assessed parenting behaviors that fell along the parenting dimension of control, with some articles measuring overcontrolling parenting behaviors that promote child avoidance (e.g., overprotection, *n* = 6), some articles measuring appropriate levels of parental control and encouragement (e.g., challenging, *n* = 3), and some articles measuring overcontrolling behaviors that excessively promote child approach (e.g., intrusiveness, *n* = 2). The remaining articles assessed dimensions related to negative and anxious affect (*n* = 4). All parenting variables were continuous except for “attribution of threat to the environment” in Murray et al. (2014), which was dichotomous. As with the temperament variables, all but one of the samples used an observation of parenting. Vreeke et al. (2013) used a self-report measure, only. Additionally, the Hudson and colleagues sample used a self-report measure in addition to an observational measure. Many articles measured more than one parenting dimension.

Three samples observed parenting behaviors during tasks in which children and parents were presented with novel stimuli (maternal encouragement to approach novelty, Kiel et al., 2016; maternal encouragement, expressed anxiety, and intrusiveness, Lawrence et al., 2020; parental dismissive, task directive, and supportive behaviors, Lorenzo et al., 2022). Two samples observed parenting behaviors during numerous structured tasks and unstructured free play (maternal overcontrol, Lewis-Morrarty et al., 2012; parental overprotective and challenging behavior, Majdandžić et al., 2018). Murray et al. (2014) observed maternal encouragement and threat attribution while reading a book about starting school. Hudson and colleagues observed maternal overinvolvement and negativity during the preparation and delivery of a speech, and had parents complete the Control scale of the Parent Protection Scale (Thomasgard et al., 1995). Vreeke et al. (2013) used the Parental Overprotection Measure, a self-report survey (Edwards, 2007). Most of the samples (*n* = 5) used more than one parenting variable, as described above. In each sample, these variables were assessed in separate moderation analyses. See Table 2 for more detail on how each article measured and defined their parenting variable, in addition to how these constructs are categorized in the current review.

**Table 2:** Parenting measures and categorization

Study	Construct	Measures and methods	Operational definition	Categorization in current review
Hudson & Dodd, 2012; Hudson et al., 2011; Hudson et al., 2019	Overinvolvement	Observed behaviors (i.e., general involvement, unsolicited help, directing child speech) during speech preparation task; Parental report of self-sacrificing and overprotective behavior during the Five-Minute Speech Sample	Parenting behavior that helps the child more than is needed and overly protects them from potential danger and distress	Control-related: promotion of child avoidance
	Negativity	Observed behaviors (i.e., general mood and atmosphere, positive affect, verbal and nonverbal encouragement, and criticism) during speech preparation task; Negative first statement, parental criticism, and report of a negative relationship during Five-Minute Speech Sample	Parenting that is characterized by more criticism and less warmth	Affect-related
Kiel et al., 2016	Encouragement to approach novelty	Observed behaviors (e.g., low scores = holding child back, verbally promoting avoidance, excessive comforting; middle score = positive verbal encouragement to approach, reinforcing play, modeling approach; high scores = physically pushing to approach, verbal demands to approach) during novelty tasks	Continuum of encouragement from no encouragement to approach (i.e., protective behavior) to behaviors that increase child independence through supporting child choice and acknowledging child perspectives (i.e., autonomy granting) to excessive encouragement (i.e., intrusive behavior)	Control-related: encouragement
Lawrence et al., 2020	Encouragement	Observed behaviors (e.g., positive comments, encouraging facial expressions and gestures, motivation to engage, enthusiasm for child effort) during novelty tasks	Not provided	Control-related: encouragement
	Expressed anxiety	Observed behaviors (e.g., biting lip, tense posture, worried expression, hand-wringing, fearful facial expression, nervous or rapid speech) during novelty tasks	Not provided	Affect-related
	Intrusiveness	Observed behaviors (e.g., speaking for child, touching infant during infant’s interaction with stranger, physical and verbal interference in child’s behavior, imposing agenda) during novelty tasks	Not provided	Control-related: excessive promotion of child approach
Lewis-Morrarty et al., 2012	Overcontrol	Observed behaviors (e.g., verbally dominating tasks and conversations, excluding child, frequent instructions, interrupting child, physically moving child away from interests, grabbing toy from child) during Hane et al., 2008 tasks	Controlling behaviors that are inappropriate and excessive relative to the child’s behavior and interests	Control-related: promotion of child avoidance
Lorenzo et al., 2022	Dismissive	Observed behaviors (e.g., asking child questions, describing what child is doing, ignoring child, being critical of child) during LabTAB episodes	Not provided	Affect-related
	Task-Directive	Observed behaviors (e.g., physically directing child, telling child what to do) during LabTAB episodes	Not provided	Control-related: excessive promotion of child approach
	Supportive	Observed behaviors (e.g., praising, suggesting strategy to reduce negative emotion, asking about child’s feelings) during LabTAB episodes	Overly solicitous, overly supportive	Control-related: promotion of child avoidance
Majdandžić et al., 2018	Challenging	Observed behaviors (e.g., chasing child, throwing baby in the air, tickling, rough-and-tumble play, challenging child to push their limits) during structured tasks and unstructured free play	Behaviors that playfully encourage child to go out of their comfort zone	Control-related: encouragement
	Overprotective	Observed behaviors (e.g., comforting child when child was not in distress, making comments about safety, handling child very carefully) during structured tasks and unstructured free play	Behaviors that communicate excessive worry and concern for child’s wellbeing and safety	Control-related: promotion of child avoidance
Murray et al., 2014	Encouragement	Recorded language (e.g., “You remember how happy your sister was when she started school,” “You are going to really enjoy school”) while reading a book about going to school to child	Communication that encourages child to engage in challenging situations	Control-related: encouragement
	Attribution of threat to the environment	Recorded language (e.g., “Those children look scary,” “There are lots of strange children in the classroom”) while reading a book about going to school to child	Communication that indicates that the environment is threatening	Affect-related
Vreeke et al., 2013	Overprotection	The Parental Overprotection Measure, a self-report rating scale with items reflecting behaviors in specific, potentially threatening situations for preschoolers (e.g., “I protect my child from criticism,” “I am reluctant for my child to play some sports for fear he/she might get hurt”)	Behaviors that shield child from potential danger by unnecessarily helping child and limiting child’s exposure to a variety of situations	Control-related: promotion of child avoidance

#### Measures of Child Anxiety

Child anxiety was assessed via clinical interviews and parent- and self-report measures across the articles. Because each Hudson and colleagues article used a different timepoint for their child anxiety measure, this sample will be broken down by article. Seven of the ten articles used a clinical interview administered by a trained researcher or clinician. Five of these seven articles also used a parent-report or child self-report measure. The remaining three articles used parent- and child-report measures only.

Five of the ten articles administered the Anxiety Disorders Interview Schedule for DSM-IV parent version (ADIS-P, Hudson & Dodd, 2012; Hudson et al., 2011, 2019; Lawrence et al., 2020; Murray et al., 2014; Silverman & Albano, 1996). In all of these articles, the ADIS-P was used to create a dichotomous value for the presence or absence of a diagnosis. Hudson and Dodd (2012) and Hudson et al. (2011) also used the ADIS-P to generate a continuous measure of number of diagnoses. Lewis-Morrarty et al. (2012) used the Kiddie Schedule for Affective Disorders and Schizophrenia for School-Age Children-Present and Lifetime Version (K-SADS-PL, Kaufman et al., 1997) with supplemental questions pulled from the Anxiety Disorder Interview Schedule for Children (Silverman & Albano, 1996) to dichotomously measure the presence or absence of a diagnosis, interviewing both adolescents and their parents separately.

Four articles used the Preschool Anxiety Scale (PAS; Spence et al., 2001) or the Preschool Anxiety Scale-Revised (PAS-R; Edwards et al., 2010b), a parent-report measure (PAS: Hudson et al., 2011, 2019; PAS-R: Majdandžić et al., 2018; Vreeke et al., 2013). Hudson et al. (2019) also used the Spence Children’s Anxiety Scale (Spence, 1998). Two articles (Lawrence et al., 2020; Lorenzo et al., 2022) used the Anxiety Problems scale of the parent-reported Child Behavior Checklist (CBCL; Achenbach & Rescorla, 2000). Lorenzo et al. (2022) also used the social anxiety subscale of the Screen for Child Anxiety Related Emotional Disorders (SCARED, Birmaher et al., 1997), a child-report measure. Lewis-Morrarty et al. (2012) used the social anxiety subscale of the SCARED, as well, with both parents and children reporting child anxiety. Kiel et al. (2016) used the separation distress subscale of the Infant Toddler Social-Emotional Assessment—Revised (ITSEA, Carter & Briggs-Gowan, 2000), a parent-report measure. All of the parent and child-report measures were continuous variables.

Five of the articles used more than one measure of child anxiety. Lorenzo et al. (2022) used the CBCL to control for earlier anxiety symptoms and the SCARED to create an unconditional growth curve model of social anxiety over time, which was used as the outcome. Hudson et al. (2019) used the PAS and the Spence Children’s Anxiety Scale to create a growth curve of anxiety over time for one analysis, and used the ADIS-IV as the outcome in a separate analysis. The remaining three articles used each of their anxiety measures as outcomes in separate analyses (Hudson et al., 2011; Lawrence et al., 2020; Lewis-Morrarty et al., 2012).

## Results

### Quality Assessment

The current review coded the included articles to assess their risk of bias using an adaptation of the Quality in Prognosis Studies (QUIPS) tool developed by Hayden et al. (2013). The QUIPS tool assesses bias in the areas of study participation, study attrition, prognostic factor measurement, outcome measurement, study confounding, and statistical analysis and reporting. A quality assessment tool was developed for the current review that assigned 0 (no risk of bias) or 1 (risk of bias) to 21 codes. See Table 3 for a complete description of the adapted scale.

**Table 3:** Adapted QUIPS tool

Code	Values
Reporter of variable^a^	0 = observation 1 = parent or other-report
Internal consistency/interrater reliability of variable^a,b^	0 = α ≥ .7, κ ≥ .8, ICC ≥ .75, *r* ≥ .80 1 = α < .7, κ < .8, ICC < .75, *r* < .80
Type of variable^a^	0 = continuous 1 = dichotomous
Adequate proportion of data available^a^	0 = ≥ 75% of data available 1 = < 75% of data available
Methodology used and setting^a^	0 = consistent across sample 1 = inconsistent across sample
Description of statistical analyses	0 = sufficient description of analyses 1 = insufficient description of analyses
Management of missing data	0 = full information maximum likelihood, multiple imputation, or expectation-maximization algorithm 1 = listwise or pairwise deletion or mean substitution
Statistical analyses	0 = adequate given study design 1 = inadequate given study design
Confounding variables	0 = potential confounds are measured and accounted for in analyses 1 = potential confounds are not measured or are not accounted for in analyses
Description of population, sample, recruitment, and inclusion and exclusion criteria	0 = sufficient descriptions 1 = insufficient descriptions
Sample maintained to follow-up	0 = ≥ 75% of sample maintained to follow-up 1 = < 75% of sample maintained to follow-up
Differences between participants who completed study versus those who were lost to follow-up	0 = no important differences 1 = important differences

When there was insufficient information to determine a value for a code, a 1 was assigned

^a^Separate codes were assigned for the temperament, parenting, and anxiety variables

^b^When there were multiple metrics for one variable, 0 was assigned if 50% or more of the metrics or the average or upper value of the metrics were above the minimum

The first and second authors each coded all of the articles. Initial agreement was calculated via kappa and percent agreement. Quality code kappas ranged from 0.78 to 1.00 across the 21 codes, indicating moderate to strong agreement (κ_mean_ = 0.96). When either coder assigned only 0s across an entire code, kappa could not be calculated. In these nine cases, percent agreement was calculated. Percent agreement of these nine codes ranged from 80 to 100% with a mean percent agreement of 95%. After coding was completed separately, the first and second author met to reach consensus on the quality codes. The 21 codes were then summed for each article to create one composite code capturing the overall risk of bias. Articles were assigned composite quality codes between 3 and 11 with a mean quality score of 5.6. Articles with scores below the mean are considered to have a low risk of bias, and articles with scores above the mean are considered to have a higher risk of bias. Findings from articles with lower risk of bias will be given greater weight given their higher quality and rigor. See Table 4 for a summary of findings.

**Table 4:** Summary of findings

Type of anxiety	Type of parenting	Significant moderation results	Non-significant moderation results
Social anxiety symptoms	Control-related: excessive promotion of child approach	None	Lorenzo et al., 2022 (2)
	Control-related: promotion of child avoidance	Lewis-Morrarty et al., 2012 Lorenzo et al., 2022 (2) Vreeke et al., 2013	Vreeke et al., 2013
	Affect-related	Lorenzo et al., 2022	Lorenzo et al., 2022
Other anxiety symptoms	Control-related: excessive promotion of child approach	None	Lawrence et al., 2020
	Control-related: promotion of child avoidance	Hudson et al., 2019	Hudson et al., 2011 Hudson et al., 2019 Majdandžić et al., 2018 (2) Vreeke et al., 2013 (2)
	Control-related: encouragement	Kiel et al., 2016 Lawrence et al., 2020	Majdandžić et al., 2018 Majdandžić et al., 2018^a^
	Affect-related	None	Hudson et al., 2011 Hudson et al., 2019 (2) Lawrence et al., 2020
Anxiety disorders	Control- and affect-related	None	Hudson & Dodd, 2012 (4) Hudson et al., 2011 (2) Hudson et al., 2019 (2) Lawrence et al., 2020 (3) Lewis-Morrarty et al., 2012^a^ Murray et al., 2014 (2)

^a^Denotes a marginally significant effect

### Social Anxiety Symptoms

Three of the articles assessed the interaction between parenting and child inhibited temperament in the prediction of social anxiety symptoms. One of these articles included three different parenting dimensions and assessed social anxiety at one timepoint and over time. Another article reported separate results by ethnicity. Therefore, nine moderation analyses were evaluated.

#### Control-Related Parenting

The majority of the moderation analyses assessing social anxiety symptoms measured a parenting dimension related to control (*n* = 7, 78%). The two analyses that assessed control-related behavior that excessively promoted child approach (e.g., intrusiveness) did not find evidence for moderation (Lorenzo et al., 2022). However, there was some evidence that control-related parenting behaviors that promote child avoidance, such as overprotection and overly supportive parenting, moderate the relation between child inhibited temperament and child social anxiety symptoms. Four of five analyses (80%) found significant moderation, with small to medium effect sizes across the analyses. Three of these four significant analyses found that the positive relation between child inhibited temperament and social anxiety symptoms was strengthened within the context of avoidance-promoting parenting behaviors (Lewis-Morrarty et al., 2012; Lorenzo et al., 2022; Vreeke et al., 2013). However, one analysis found that high child inhibited temperament predicted a sharper reduction in social anxiety symptoms over time within the context of high avoidance-promoting parenting behaviors (Lorenzo et al., 2022). Thus, the impact of these parenting behaviors may be nuanced based on whether anxiety is assessed at one time point (or as change between time points) versus as a trajectory over time. Notably, the one sample with a rigorous design and low risk of bias (Lewis-Morrarty et al., 2012; quality code = 3) found overcontrol to be a significant moderator of medium effect size.

The control-related parenting moderation findings were supported by analyses with varying methods for measuring temperament, parenting, and anxiety (parent-report, observation) and varying child ages at anxiety measurement (childhood through adolescence). Almost all of the studies included parent–child dyads and did not recruit mothers or fathers in particular, except for Lewis-Morrarty et al. (2012), who only recruited mothers. Further, all of the control-related parenting analyses strengthened the rigor of their moderation by noting that child inhibited temperament did not significantly relate to parenting behavior.

#### Affect-Related Parenting

There was not enough evidence in the literature to determine whether affect-related parenting moderates the relation between child inhibited temperament and child social anxiety symptoms, with only one study with two analyses in the review (Lorenzo et al., 2022). One of these analyses found that dismissive parenting moderated the relation between child inhibited temperament and change in social anxiety symptoms over time, with high dismissive parenting strengthening the relation between high inhibited temperament and stability in social anxiety symptoms. However, there was no evidence of moderation when social anxiety was assessed at one timepoint.

### Other Anxiety Symptoms

Across the six articles that investigated other anxiety symptoms, such as total anxiety, separation anxiety, and non-social anxiety symptoms, there was a different pattern of findings. Four of these articles included more than one parenting dimension (Hudson et al., 2011, 2019; Lawrence et al., 2020; Majdandžić et al., 2018), one article divided the sample based on ethnicity (Vreeke et al., 2013), one article assessed anxiety both at one timepoint and over time (Hudson et al., 2019), and one article divided their analyses between mothers and fathers (Majdandžić et al., 2018). Therefore, sixteen moderation analyses were reviewed.

#### Control-Related Parenting

The majority of moderation analyses assessing other anxiety symptoms as the outcome measured a parenting dimension related to control (*n* = 12, 75%), altogether yielding mixed evidence. One analysis assessed parenting behavior that excessively promoted child approach, and found no evidence of moderation (Lawrence et al., 2020). However, there was mixed evidence for parenting behaviors that promote child avoidance. Six of the seven analyses (86%) that assessed parenting behaviors that promote child avoidance found non-significant results (Hudson et al., 2011, 2019; Majdandžić et al., 2018; Vreeke et al., 2013). However, when only high-quality studies were reviewed (Hudson et al., 2011, 2019), there was one significant finding and two non-significant findings. Notably, Hudson et al. (2019) was the only analysis with overprotection that assessed child through adolescent anxiety as the outcome in addition to child anxiety at one timepoint. Given that Hudson et al. (2019) was also the only study to find a significant moderation, it is possible that avoidance-promoting parenting behaviors only moderate the relation between inhibited temperament and the trajectory of child anxiety symptoms over time.

There was some evidence for parental encouragement moderating the relation between inhibited temperament and early childhood anxiety symptoms. Two of four analyses (50%) found that parental encouragement moderated the relation between inhibited temperament and other anxiety symptoms (Kiel et al., 2016; Lawrence et al., 2020). However, the directionality of this effect differed. Lawrence et al. (2020) found that encouraging parenting was an adaptive parenting behavior that led to better anxiety outcomes within the context of stable inhibited temperament. Conversely, Kiel et al. (2016), a high-quality study (quality score = 3) found that encouragement served as an adaptive parenting behavior that led to better separation anxiety outcomes in children with low inhibited temperament, but served as a maladaptive parenting behavior that led to worse separation anxiety outcomes in children with very high inhibited temperament. There were no notable differences in measurement, sample, type of anxiety measured (separation anxiety vs. total anxiety), and child age between the significant and non-significant analyses. All of the studies assessed child anxiety in early childhood (age 2–5 years). Further research is needed to clarify whether encouraging parenting contextualizes a positive or negative relation between child inhibited temperament and child anxiety.

#### Affect-Related Parenting

There was a lack of evidence that affect-related parenting, namely negativity and expressed anxiety, moderates the relation between inhibited temperament and non-social anxiety symptoms. Three articles assessed affective parenting behaviors as a moderator with a total of four analyses. All four analyses (100%) had non-significant results (Hudson et al., 2011, 2019; Lawrence et al., 2020). Notably, all three of these studies recruited parents and children from the community and pre-screened them for inclusion based on particular characteristics (e.g., children with high and low levels of behavioral inhibition, mothers with and without anxiety disorders). Thus, more research is needed in community samples without pre-screening inclusion criteria. However, the consistent lack of evidence found for this moderation across these analyses suggests that negative and anxious parenting behaviors do not moderate the relation between child inhibited temperament and child anxiety symptoms.

### Anxiety Disorders

There was a consistent lack of evidence that parenting behaviors moderate the relation between child inhibited temperament and child anxiety disorders. Six studies investigated parenting as a moderator of the relation between child inhibited temperament and child anxiety disorders. One study assessed for the presence of an anxiety disorder diagnosis and the number of anxiety disorder diagnoses (Hudson & Dodd, 2012). Five studies assessed more than one parenting behavior (Hudson & Dodd, 2012; Hudson et al., 2011, 2019; Lawrence et al., 2020; Murray et al., 2014). Therefore, fourteen moderation analyses were reviewed. Eight analyses (57%) assessed anxiety disorders generally (Hudson & Dodd, 2012; Hudson et al., 2011, 2019) and six analyses (43%) assessed social anxiety disorder in particular (Lawrence et al., 2020; Lewis-Morrarty et al., 2012; Murray et al., 2014).

Thirteen of the fourteen included analyses (93%) yielded non-significant findings (Hudson & Dodd, 2012; Hudson et al., 2011, 2019; Lawrence et al., 2020; Murray et al., 2014). The one other analysis, conducted by Lewis-Morrarty et al. (2012), found that maternal overcontrol only marginally moderated the relation between child inhibited temperament and lifetime social anxiety disorder diagnoses, and therefore, the interaction was not probed for simple effects. These fourteen analyses included assessment of the current or lifetime presence of social anxiety disorder and the presence and number of any current anxiety disorders. The analyses also varied in how temperament, parenting, and anxiety disorders were assessed, the type of parenting measure used, and the age of children when the disorders were assessed. Overall, these findings indicate that no parenting behaviors moderate the relation between child inhibited temperament and anxiety disorders.

## Discussion

The current review systematically assessed the previous literature to determine whether there is adequate evidence that parenting moderates the relation between child inhibited temperament and child anxiety. Ten articles were identified in the literature, with 39 moderation analyses among them. All of these studies took place in Western contexts with predominately non-Hispanic, White, middle-class families. Therefore, this review only reflects the moderating role of parenting in predominately White and middle-class families in Western regions, and thus may not be generalizable to all racial and ethnic groups and non-Western contexts. The current review assessed numerous characteristics of the included analyses, most notably the type of anxiety measured and the type of parenting measured. Evidence for the moderation of interest differed across the different types of anxiety and parenting assessed.

### Summary of Findings

The current review revealed that avoidance-promoting parenting behaviors such as overprotection moderate the relation between child inhibited temperament and social anxiety symptoms such that higher child inhibited temperament predicts greater social anxiety symptoms within the context of high levels of avoidance-promoting parenting behaviors. This research evidence supports developmental psychopathology theories of anxiety development that posit that children’s environments (e.g., parenting) interact with their predisposing characteristics (e.g., inhibited temperaments) to predict anxiety outcomes (Muris et al., 2011; Vasey & Dadds, 2001). Notably, the current review did not find substantial evidence for avoidance-promoting parenting behaviors moderating the relation between child inhibited temperament and other anxiety symptoms, such as total anxiety symptoms and separation anxiety symptoms. The stronger evidence for parenting moderating the relation between inhibited temperament and social anxiety, in particular, is in line with previous theory and research that links inhibited temperament to social anxiety specifically (Pérez-Edgar & Guyer, 2014).

Further, inhibited temperament captures withdrawal and hesitancy in various novel environments and contexts, some of which are social (e.g., unfamiliar person) and some of which are non-social (e.g., unfamiliar toy). There is evidence that inhibited temperament assessed in social contexts (i.e., social behavioral inhibition) relates differently to anxiety outcomes than inhibited temperament assessed in non-social contexts (i.e., non-social behavioral inhibition; Dyson et al., 2011; Tan et al., 2024). It may be that social inhibition in particular is driving the relation between inhibited temperament and anxiety, thus explaining the unique relation between inhibition and social anxiety that is further strengthened in the presence of anxiogenic parenting behaviors. It may also be that parenting that promotes child avoidance plays a role in the development of social anxiety, specifically. When parents engage in protective and overly supportive parenting, children with higher inhibited temperament are encouraged to avoid rather than engage with novel social environments, reinforcing the use of social withdrawal as a coping strategy and strengthening the belief that they cannot independently navigate the social world.

The finding that avoidance-promoting parenting behaviors are particularly important risk factors when combined with child inhibited temperament supports the anxious-coercive family systems theory that emphasizes the role of overcontrolling and overprotective parenting in particular in anxiety development (Dadds & Roth, 2001). As suggested by the anxious-coercive cycle, there may be a direct relation between temperament and parenting in which inhibited children elicit overprotective parenting behaviors. However, the current review indicates that when examining anxiety outcomes over time, avoidance-promoting parenting appears to moderate this relation. Further research is needed to tease apart the interaction between avoidance-promoting parenting and inhibited temperament in the prediction of social anxiety in particular to determine why this specificity exists.

When considering non-social anxiety, the current review revealed some evidence that encouraging parenting behaviors moderate the relation between child inhibited temperament and other anxiety symptoms; however, the direction of this relation within the context of encouraging parenting remains unclear. The way in which encouragement was measured in the included studies may have impacted the difference in the directionality of this effect. Lawrence et al. (2020) assessed gentle and enthusiastic encouragement only, whereas Kiel et al. (2016) assessed a continuum of encouragement with low values representing excessive comforting, middle values representing gentle encouragement, and high values representing excessive encouragement and intrusiveness. It may be that gentle encouragement is adaptive for children with high inhibited temperaments, whereas excessive encouragement is maladaptive for these children. Further research assessing various levels and types of encouragement will be helpful for elucidating this moderating effect.

Study findings indicated that parenting defined by negative affect does not moderate the relation between child inhibited temperament and other anxiety symptoms. The lack of evidence for parenting defined by negative affect moderating the relation between inhibition and anxiety is consistent with theory that focuses on the role of control-related parenting behaviors specifically in anxiety development (Chorpita & Barlow, 1998; Dadds & Roth, 2001). It may also be that affect-related parenting behavior predicts anxiety development across levels of child temperament, instead of interacting with inhibition to predict anxiety outcomes. Further research is needed to clarify whether parenting defined by negative affect moderates the relation between child inhibited temperament and social anxiety symptoms, given the limited research assessing this interaction with social anxiety specifically as the outcome.

Lastly, the current review revealed that no parenting behaviors moderated the relation between child inhibited temperament and whether or not children were diagnosed with anxiety disorders. The consistent lack of evidence for this moderation may be due to various factors. First, it is more difficult to find significant effects for dichotomous outcomes and outcome measures with low variance. Thus, it was statistically more difficult to find an effect when outcomes were the presence or absence of an anxiety disorder or the number of anxiety disorders as compared to when outcomes were anxiety symptoms. Second, anxiety diagnoses are given based on meeting a certain amount of specific diagnostic criteria, thus creating a cutoff for anxiety symptoms. It may be that there were many children with subclinical symptoms in the non-anxiety disorder groups and many children with low levels of clinical symptoms in the anxiety disorder groups that were not qualitatively distinct from one another, and actually had similar levels of anxiety symptoms. Therefore, it may be that these diagnostic cutoffs masked potential moderating effects. Lastly, it may be that the relation between inhibition and anxiety disorder diagnoses is indeed not impacted by the contextual role of parenting, and operates independently of the parenting children receive.

### Clinical Implications

The current review found some evidence that overcontrolling and overprotective parenting behaviors moderate the relation between child inhibited temperament and child social anxiety with a small to medium effect size. This finding suggests that prevention and intervention efforts should focus on decreasing parenting behaviors that promote child avoidance, in particular. Additionally, these interventions may be most important for children with high inhibited temperaments. There are numerous parenting interventions for child anxiety that are efficacious (Comer et al., 2019; Lebowitz et al., 2020; Smith et al., 2014), some of which target children with high inhibited temperament in particular (Bayer et al., 2011; Rapee, 2013). The current review provides support for implementing these interventions that target parenting behaviors, given evidence that without these avoidance-promoting behaviors, the positive relation between inhibited temperament and social anxiety symptoms may be weakened.

### Limitations of the Literature and Future Directions

There are various limitations of the current literature that highlight necessary future directions for the field. Primarily, the literature that prospectively assesses parenting as a moderator solely exists in Western countries with predominately White and middle-class samples. Therefore, the findings from the literature cannot be generalized to non-White or low-income individuals, and does not apply to non-Western contexts. Substantial research is needed in these samples in order to provide insight into how child temperament and parenting interact in the trajectory to anxiety development in these populations. Further, culture is complex and nuanced and cannot be captured in its entirety by quantitative measures of demographic characteristics such as race, ethnicity, or socioeconomic status. For instance, a child’s neighborhood, school, peers, and family environment all play a role in their cultural values, beliefs, and context. Future quantitative and qualitative research investigating specific cultural factors and their relation to this moderation are needed.

There were also methodological limitations to the literature. The studies in the current review had decent sample sizes ranging from 117 to 293 participants. However, it is difficult to find significant effects in longitudinal studies, and even more difficult to find significant interaction effects longitudinally. Therefore, it may be that many of these studies did not have sufficient power to detect an interaction effect. Further research with larger sample sizes will strengthen the evidence for this moderation and help determine the size of its effect. It is also important to consider the methodological rigor and the quality of included studies. Half of the included studies were considered high quality with low risk of bias across the areas of participants, measures, statistics, and reporting, whereas the other half were considered to have a higher risk of bias and lower quality. It may be that the findings of the current review would differ if there were more studies with low risk of bias. For example, the findings for control-related parenting behaviors moderating the relation between inhibition and other anxiety symptoms became more ambiguous when only high-quality studies were considered. Therefore, further high-quality studies with rigorous methodology and reporting are needed to further elucidate this moderation. Some considerations for improving the quality and rigor of studies include reporting the correlation between child temperament and parenting behavior and assessing for differences between participants who were retained and those who were lost to follow-up. Four of the ten included articles in the current review did not report these correlations, and therefore, it cannot be confirmed that their analyses conformed to the typical assumption of moderation analyses. Additionally, four of the ten included articles did not assess for differences between these participants.

The literature is also limited by the way in which parenting is measured. Most of the included articles only assessed parenting at one time point using one method (i.e., observation only, self-report only). Additionally, many of the observational measures were brief in nature (e.g., 3 min). Multimethod assessment for longer periods of time and at multiple timepoints might better capture caregivers’ parenting behaviors. Further, there is minimal research on the stability of parenting behaviors across child development (Verhoeven et al., 2007). If parenting behaviors change across development, then results regarding parenting as a moderator of the relation between inhibited temperament and child anxiety may also differ if parenting is measured over time versus at one timepoint. Future research would benefit from more comprehensive, longitudinal assessments of parenting behavior.

An additional limitation in the current literature is a primary focus on mothers. Seven of the ten included articles assessed maternal parenting behavior only. Although mothers are important caregivers to investigate, there are many other caregivers who have an important impact on child development. Future research should assess the moderation of interest in alternative caregivers, such as fathers, grandparents, and foster parents to elucidate the trajectory to child anxiety development in these families.

Another limitation of the literature is an emphasis on a diathesis-stress framework for understanding the roles of child inhibited temperament and parenting behavior. Most of the articles in the current review (nine out of ten) studied the moderation analysis within the context of a diathesis-stress model. In other words, these nine studies considered inhibited temperament to be a risk factor for worse anxiety outcomes when in the presence of maladaptive parenting behaviors. Only one study, Majdandžić et al. (2018), also considered that inhibited temperament may function within alternative moderation models in addition to a diathesis-stress model, such as a vantage-sensitivity or differential susceptibility model. Notably, Majdandžić et al. (2018) were one of only three articles that assessed a form of parental encouragement, which has been shown to serve an adaptive function for children with high inhibited temperaments (McLeod et al., 2007). Given that most of the articles in this review operated within a diathesis-stress framework, the potential role of child inhibited temperament as a protective or susceptibility factor may have been overlooked and thus not adequately assessed. Future research should consider these alternative models and assess the moderating role of adaptive parenting behaviors, in addition to maladaptive parenting behaviors, in the relation between child inhibited temperament and child anxiety.

The literature would also benefit from more comprehensive models of the developmental pathway to child anxiety, including other known correlates of inhibited temperament and anxiogenic parenting behavior, such as parental anxiety and cognitions (Borelli et al., 2015; Feinberg et al., 2018; Jones et al., 2021). Research has indicated that mothers with higher anxiety engage in more overprotective parenting behavior (Jones et al., 2021) and that parental negative beliefs about child anxiety relate to anxiogenic parenting behavior (Feinberg et al., 2018). Further, anxious parents are more likely to have children with anxiety, suggesting genetic and biological correlates that may influence the developmental pathway to child anxiety (Perlman et al., 2022). Future research with larger samples could examine a larger model in which child inhibited temperament and parental anxiety, beliefs, and behaviors are all included as contributors to child anxiety development.

The final limitation in the current literature is the relatively small number of studies that assess parenting as a moderator in the longitudinal relation between inhibited temperament and child anxiety. The current review assessed all relevant research, and only ten articles met criteria for inclusion in the review. Further research assessing affect- and control-related parenting behaviors as moderators in the relation between inhibited temperament and anxiety is needed to confirm the findings of the current review and expand upon the current understanding of this moderation.

### Limitations of the Current Review

The current review is impacted by various limitations. First, the current review only assessed published journal articles, and thus did not include dissertations or unpublished results. Given the publication bias toward significant findings, the evidence found for avoidance-promoting parenting behaviors moderating the relation between child inhibited temperament and child social anxiety with a small to medium effect may be weaker than suggested in the current review. Second, the current review solely assessed study findings qualitatively. Future meta-analytic research may provide more quantitative insight into the strength of this moderation effect and how the effect differs across various demographic and sample characteristics. Third, the current review included three articles that used the same sample. Thus, this sample was overrepresented, and it is possible that there are unique characteristics of this sample that were overrepresented in the findings of the current review.

### Conclusion

The current review systematically assessed the strength of the evidence for parenting moderating the relation between child inhibited temperament and child anxiety. There was some evidence that overprotective and overcontrolling parenting behaviors moderate the relation between child inhibited temperament and child social anxiety symptoms with a small to medium effect size. There was a consistent lack of evidence that parenting moderated the relation between child inhibited temperament and anxiety disorders and that parental negativity and expressed anxiety moderated the relation between child inhibited temperament and non-social anxiety symptoms. There was mixed evidence regarding the moderating role of control-related parenting behaviors in the relation between child inhibited temperament and non-social anxiety symptoms. Future research is needed to clarify these nuanced and inconsistent findings.
